# *TLR9 *polymorphisms in African populations: no association with severe malaria, but evidence of *cis*-variants acting on gene expression

**DOI:** 10.1186/1475-2875-8-44

**Published:** 2009-03-13

**Authors:** Susana Campino, Julian Forton, Sarah Auburn, Andrew Fry, Mahamadou Diakite, Anna Richardson, Jeremy Hull, Muminatou Jallow, Fatou Sisay-Joof, Margaret Pinder, Malcolm E Molyneux, Terrie E Taylor, Kirk Rockett, Taane G Clark, Dominic P Kwiatkowski

**Affiliations:** 1 Wellcome Trust Centre for Human Genetics, University of Oxford, Roosevelt Drive, Oxford, OX3 7BN, UK; 2 Wellcome Trust Sanger Institute, Hinxton, Cambridge, CB10 1SA, UK; 3 University Department of Paediatrics, John Radcliffe Hospital, Oxford, OX3 9DU, UK; 4 Medical Research Council Laboratories, Atlantic Boulevard, Fajara, PO Box 273, Banjul, the Gambia; 5 Malawi-Liverpool-Wellcome Programme of Clinical Tropical Research, College of Medicine, Blantyre, PO Box 30096, Malawi; 6 Blantyre Malaria Project, College of Medicine, Blantyre, PO Box 30096, Malawi

## Abstract

**Background:**

During malaria infection the Toll-like receptor 9 (*TLR9*) is activated through induction with plasmodium DNA or another malaria motif not yet identified. Although *TLR9 *activation by malaria parasites is well reported, the implication to the susceptibility to severe malaria is not clear. The aim of this study was to assess the contribution of genetic variation at *TLR9 *to severe malaria.

**Methods:**

This study explores the contribution of *TLR9 *genetic variants to severe malaria using two approaches. First, an association study of four common single nucleotide polymorphisms was performed on both family- and population-based studies from Malawian and Gambian populations (n>6000 individual). Subsequently, it was assessed whether *TLR9 *expression is affected by *cis*-acting variants and if these variants could be mapped. For this work, an allele specific expression (ASE) assay on a panel of HapMap cell lines was carried out.

**Results:**

No convincing association was found with polymorphisms in *TLR9 *for malaria severity, in either Gambian or Malawian populations, using both case-control and family based study designs. Using an allele specific expression assay it was observed that *TLR9 *expression is affected by *cis*-acting variants, these results were replicated in a second experiment using biological replicates.

**Conclusion:**

By using the largest cohorts analysed to date, as well as a standardized phenotype definition and study design, no association of *TLR9 *genetic variants with severe malaria was found. This analysis considered all common variants in the region, but it is remains possible that there are rare variants with association signals. This report also shows that *TLR9 *expression is potentially modulated through *cis*-regulatory variants, which may lead to differential inflammatory responses to infection between individuals.

## Background

Exploring the idea that malaria motifs can induce the innate immune system and initiate an inflammatory response may lead to a greater understanding of resistance/susceptibility to severe malaria. Several reports have found that malaria parasites express molecules that are recognized by Toll-like receptors (TLR), which play a critical role in the early innate immune response to invading pathogens. For instance, the malaria pigment haemozoin has been shown to function as carrier for plasmodium DNA which stimulates *TLR9 *and induces inflammation [1-3]. Activation of *TLR9 *expressed on dendritic cell has also been proposed as a mechanism used by malaria parasites to trigger regulatory T cells and evade the immune system [4]. Regulatory T cells were also shown to contribute to the pathogenesis of severe malaria by suppressing anti-malarial immunity during *Plasmodium berghei *ANKA infection [5]. The correct balance between induction of an immune response against the malaria parasite and limitation of suppression of host immunity by the parasite seems essential to control infection.

To assess the clinical relevance of *TLR9 *polymorphisms and severe malaria two association studies were conducted in Ghana. While one of the studies did not observe any clear association with severe malaria[6], another found *TLR9 *polymorphisms to be associated with the clinical manifestation of malaria during pregnancy [7]. Recently, the TLR-9-1486C/T variants was found to be associated with high parasitaemia in a cohort of patients with mild malaria from the Amazonian region of Brazil [8].

This report explores the contribution of *TLR9 *genetic variants to severe malaria using two approaches. At first, a large case-control and family-based association studies was performed in Malawian and Gambian populations using four common polymorphisms located in the *TLR9 *gene region. It is also known that distant SNPs can affect gene expression, and these can only be identified using functional *in vitro *assays [9-11]. Therefore, subsequently it was assessed whether *TLR9 *expression is affected by *cis*-acting variants and whether these variants could be mapped. Such polymorphism can be further investigated as candidates for possible association with severe malaria. Exploiting the fact that *TLR9 *is a major TLR expressed by human B cells [12], an allele specific expression (ASE) assay was performed using HapMap [13] B-lymphoblastoid cell lines (LCLs), for which genotypes on hundreds-of-thousands SNPs exist, permitting the correlation between expression data and genetic variability [11,14-17] and thus facilitating the discovery of putative functional polymorphisms.

## Methods

### Study participants

Patient samples were collected as part of ongoing epidemiological studies of severe malaria at the Royal Victoria Hospital, Banjul, The Gambia (885 severe malaria cases, 629 controls; 1,044 family trios) and the Queen Elizabeth Central Hospital, Blantyre, Malawi (712 severe malaria cases, 416 controls; 248 family trios). The set of nuclear family trios comprise a severe malaria affected child and its two (biological) parents, and were assessed as 'true trios' using the *Nuclear *software package[18]. All DNA samples were collected and genotyped following approval from the relevant research ethics committees and informed consent from participants.

### Phenotypic definition

All cases were children admitted to hospital with evidence of *Plasmodium falciparum *on blood film and clinical features of severe malaria [19]. Subjects were defined as having had 'cerebral malaria' if their Blantyre coma score was less than or equal to 3 on presentation or early during admission. A second phenotypic subset of 'severe malarial anaemia' was defined as those subjects having had a haemoglobin density of less than 5 g/dl or a haematocrit less than 15%. Participants with co-existing severe or chronic medical conditions (e.g. bacterial pneumonia, kwashiorkor) unrelated to a severe malarial infection were excluded. Controls were cord blood samples obtained from birth clinics in the same locality as the cases, and thought to approximate a random sample of the population thus, reflecting the true allele frequency. Cerebral malaria (CM) and severe malarial anaemia (SMA) were the most common symptoms defining severe malaria.

### Sample preparation and genotyping

For the association studies (participants described below) genomic DNA samples underwent whole genome amplification through either Primer Extension Pre-amplification [20] or Multiple Displacement Amplification [21], before genotyping on a Sequenom MassArray genotyping platform. Only the four common SNPs (minor allele frequency (MAF) of at least 5% in the HapMap Yoruba population) in the *TLR9 *region were genotyped. The majority of polymorphisms in this region are monomorphic.

For the ASE assay 19 unrelated CEU and 20 YRI individuals were used, selected from the HapMap collection. Established lymphoblastoid cell lines (LCLs) from these individuals were obtained from the Centre d'Etude du Polymorphisme Humain (CEPH) collection (Coriell Institute for Medical Research). Cell lines were cultured has described previously [22].

### Expression phenotyping

For expression analysis, total RNA was extracted from cells lines from each individual using Tri reagent (Sigma). Poly A+ RNA was isolated from total RNA using the Dynabeads mRNA Purification Kit (Dynal). Synthesis of First-Strand cDNA was processed according to the StrataScript™ First-Strand Synthesis System (Stratagene). The Allelotype platform from Massarray (Sequenom) was utilized for accurate relative quantification of allele specific cDNA and gDNA transcripts [23]. Primers were designed using the dedicated software Spectrodesigner (Sequenom). Allele specific expression quantification was performed according to the protocol of Forton *et al *[15] and Campino *et a*l [24]. Briefly, for each cDNA and gDNA assays, nine technical replicates were performed. Biological replicates from independent cultures were assayed equally. For a given cDNA or gDNA assay the allelic transcript ratio was calculated on each of the nine technical replicates from the relative quantity of the two allele-specific transcripts. The mean allelic transcript ratio for the whole assay was then calculated, and normalized to the mean allelic transcript ratio for the genomic controls. An assay was accepted for further analysis if the standard error of the mean for the technical and biological replicates was less than 10%.

### HAPMAP haplotypes

Haplotypes were downloaded from the HapMap Phase II database . For data analysis, 770,394 markers were used; all monomorphic markers were excluded from the analysis. Locations of the SNP markers were based on those of the human reference sequence  of May 2004 (hg 17, build 35).

### Statistical analysis of the case-control and family trio association studies

Genotypic deviations from Hardy-Weinberg equilibrium (HWE) were assessed using a chi-square statistical test. Case-control association analysis was undertaken by logistic regression and included the covariates: ethnic group, gender and the HbS polymorphism. Family-based association analysis was performed using the transmission disequilibrium test (TDT) [25], as well as a case-pseudo-control approach with conditional logistic regression [26]. The TDT test is robust to the effects of population stratification or sub-structure. All analysis was performed using the R statistical package.

### Statistical analysis of allelic expression

For the association analysis, log-transformed ratios of allele expression values for individuals with the heterozygous genotype at the exonic SNP (rs352140) were used. To map putative *cis*-variants a statistical analysis approach was used, based on the linear regression method (similar to that developed by Teare *et al *and Campino *et al*)[24,27]. In particular, it was assessed whether there is evidence of an increasing or decreasing (linear) relationship between the ratio of expressions for the phase-known heterozygous type 1 (e.g. AG), homozygous types (e.g. AA and GG), and heterozygous type 2 (e.g. GA) genotypes. The rationale for the approach is that allelic imbalances would be observed only in individuals heterozygous for the *cis*-acting polymorphism. All analysis was performed using the R statistical package.

## Results and discussion

### Association analyses of common *TLR9 *SNPs and severe malaria

To assess the contribution *TLR9 *polymorphisms to severe malaria susceptibility/resistance an association analysis was performed using case-control and trio cohorts from Malawian (712 cases, 416 controls; 248 family trios) and The Gambian (885 cases, 629 controls; 1,044 family trios). Cerebral malaria and severe anaemia were the most common symptoms defining severe malaria. The majority of the Gambian (70%) and Malawian (91%) cohorts comprise cases of cerebral malaria.

**Figure 1:**
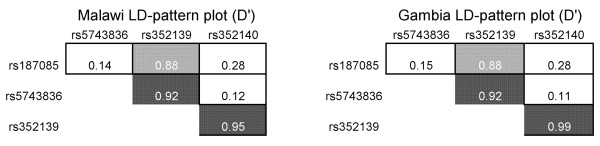
***Linkage disequilibrium *patterns between *TLR9 *polymorphisms in Malawian and Gambian cohorts**. Pairwise linkage disequilibrium between the four *TLR9 *polymorphisms was calculated using the normalized disequilibrium coefficient (D').

Of the four common SNPs in the *TLR9 *region genotyped in the Malawian and Gambian cohorts, two were located in the promoter (-1486 T>C rs187084, -1237 C>T rs5743836), one was intronic (+1174 A>G rs352139) and the last was a synonymous SNP located on exon 2 (+2848 G>A rs352140). The observed MAFs observed in the cohorts are presented in Table 1. There is no evidence of distortion from Hardy-Weinberg equilibrium (HWE) in the controls or parents (P > 0.05). There is a high degree of concordance of the allele frequencies within country and between the two study designs. The intronic SNP (+1174-X) is in high linkage disequilibrium (LD) with the other SNPs, especially with the exonic SNP (+2848-X) with which it shows a near complete LD (D' = 0.95 in the Malawian cohort and 0.99 in the Gambian) (Figure 1).

**Table 1:** Allelic frequencies of *TLR9 *polymorphisms

		Gambia		Malawi	
		Controls^1^	Cases	Controls^1^	Cases
*Case-control (n)*	*allele*	*628*	*883*	*416*	*712*
*TLR9*_rs187084	C	0.231	0.227	0.270	0.232
*TLR9*_rs5743836	C	0.460	0.438	0.407	0.381
*TLR9*_rs352139	A	0.395	0.413	0.396	0.444
*TLR9*_rs352140_exonic	T	0.244	0.249	0.302	0.263
					
*Trios (n)*		*2086*	*1043*	*376*	*188*
*TLR9*_rs187084	C	0.237	0.236	0.269	0.266
*TLR9*_rs5743836	C	0.481	0.478	0.406	0.399
*TLR9*_rs352139	A	0.435	0.418	0.378	0.391
*TLR9*_rs352140_exonic	T	0.248	0.249	0.323	0.321

^1 ^For the trios' cohort the parents were used as controls.

The allele frequencies between the Gambian cases and controls and among the parents and children from the trios are very similar (odds ratios (OR) close to 1 in both studies) (see Table 2). In the Malawian case-control cohort we observed a weak association between allele 1174-A and severe malaria (OR = 1.21, 95% CI 1.01 – 1.45; p = 0.04). The allele 2848-T showed a marginal protective effect (OR = 0.82, 95% CI 0.68 – 1.00; p = 0.05). The + 1174 and +2848 SNPs are in near perfect LD (D' close to one) and only three possible haplotypes exist (1174-A/2848-C, 1174-G/2848-C, 1174-G/2848-T). The relative risk of disease (95% CI) for haplotypes 1174-G/2848-C, 1174-G/2848-T and 1174-A/2848-C are 0.95 (95% CI: 1.01–1.33), 0.86 (95% CI 0.80–1.11) and 1.15 (95% CI: 0.73–1.03) with p-values of 0.05, 0.45 and 0.10 respectively. However the effect of these polymorphisms was not reproducible in the trios, where allele frequencies between parents and children were comparable, and there was no evidence of transmission distortion and association (Table 2). In the case-control analyses, the allelic tests do not adjust for any confounding effects, such as population substructure, that can affect bias estimates in case-control study designs. In the genotype analysis of the case-controls (Table 3), adjusted for the HbS sickle cell polymorphism, and also ethnicity in the Gambian study, no strong signals of genotypic association was found for any of the four polymorphisms.

**Table 2:** Severe malaria and *TLR9 *allelic-based association analysis

		The Gambia				Malawi			
Case-control	alleles	OR^1^	95% CI^2^		p-value^3^	OR	95% CI		p-value^3^

rs187084	C vs T	0.98	0.82	1.16	0.81	0.82	0.66	1.01	0.06
rs5743836	C vs T	0.92	0.78	1.07	0.26	0.90	0.75	1.08	0.26
rs352139	A vs G	1.08	0.93	1.25	0.33	1.21	1.01	1.45	0.04
rs352140	T vs C	1.03	0.87	1.21	0.77	0.82	0.68	1.00	0.05

Trios									

rs187084	C vs T	0.95	0.82	1.11	0.55	0.91	0.66	1.26	0.56
rs5743836	C vs T	0.95	0.78	1.16	0.62	0.82	0.59	1.16	0.26
rs352139	A vs G	0.92	0.82	1.05	0.22	1.07	0.77	1.49	0.67
rs352140	T vs C	1.00	0.87	1.16	0.97	0.99	0.72	1.36	0.94

^1^OR = odds ratio,

^2 ^CI = confidence interval

^3 ^for the trios these are TDT p-values

**Table 3:** Severe malaria and *TLR9 *genotype-based association analysis

		The Gambia^1^				Malawi^2^			
Case-control	genotypes	OR	95% CI		p-value	OR	95% CI		p-value

rs187084	CT vs TT	0.96	0.75	1.23	0.79	0.82	0.62	1.09	0.17
	CC v TT	0.98	0.57	1.70	0.97	0.60	0.35	1.02	0.06
rs5743836	CT vs TT	0.98	0.74	1.30	0.90	1.01	0.75	1.34	0.95
	CC vs TT	0.81	0.56	1.15	0.25	0.63	0.42	0.97	0.04
rs352139	AG vs GG	1.01	0.77	1.30	0.97	1.22	0.91	1.63	0.17
	AA vs GG	1.22	0.85	1.74	0.27	1.43	0.98	2.07	0.06
rs352140	TC vs CC	0.97	0.76	1.24	0.84	0.77	0.59	1.01	0.06
	TT vs CC	1.36	0.82	2.26	0.22	0.78	0.49	1.25	0.31

^1^Adjusted for HbS and ethnicity

^2 ^Adjusted for HbS

The analysis of the Gambia case-control cohorts showed that there was no evidence of association between severe malaria and the polymorphisms studied. Similarly for the Malawi case-controls, there was no strong evidence of association, but there are some marginal effects consistent with the haplotype and allelic analyses above. Accounting for multiple testing would make us less likely to believe the marginal evidence (P ≈ 0.05) of associations for some of the polymorphisms and potential haplotype effects. Mantel-Haenszel pooled estimates of risk for each SNP across the populations and studies did not lead to significant results (p > 0.05). In conclusion, no convincing association was found with polymorphisms at *TLR9 *for malaria severity, in either Gambian or Malawian populations, using both case-control and family based study designs

### Exploring the existence of functional polymorphism affecting *TLR9 *allelic expression

Candidate gene genetic studies are generally restricted to SNPs covering the gene area as potential regulatory SNPs localized away from a gene are difficult to identify. Therefore, as no association was found with SNPs in the *TLR9 *gene area, there was further exploration into whether other SNPs which can be far away from the gene could have an important role on the regulation of *TLR9 *expression.

Numerous studies have recently contributed to the identification of putative regulatory variants by correlating total gene expression or allele specific expression data with genetic variability[11,16,17,28] Using a similar approach, in this report an allele specific expression (ASE) assay was performed on a panel of HapMap B-lymphoblastoid cell lines (LCL) for which genotyped data is available and facilitates the mapping of functional polymorphisms. These cell lines have been widely used to map *cis*/*trans *regulatory regions and recent publications have shown high consistence between *cis*-regulation results obtained using HapMap cell lines and primary tissue[29].

The strength of the ASE assay is the possibility to compare the relative expression of the two alleles in heterozygous individuals within the same cellular sample. Therefore, both alleles will be exposed to the same environmental, technical and genetic factors (e.g. *trans*-acting effect). If the expression of the alleles is not equal (allelic imbalance), it suggests that the expression of the gene is under *cis-*regulation.

Cell lines from individuals of northern and western Europe origin (CEU) and from Yoruba of Ibadan, Nigeria (YRI) were selected from the HapMap collection [13] and were genotyped for a exonic synonymous SNP present on the *TLR9 *gene. Since this SNP is located within a part of the gene that is transcribed, it is possible to discriminate between allelic transcripts in heterozygous individuals.

Twenty-one unrelated CEU and nineteen YRI individuals were found to be heterozygous for this SNP and were used for the ASE analysis. For these individuals, their genomic DNA was used to obtain the reference equi-molar ratio between the two alleles (allele1 and allele2), and this was then compared with their allelic RNA expression. Allelic expression imbalance (AEI) was determined if independent replicate assays in the RNA samples showed differential allele expression ratios that deviated from the genomic DNA. If there is no allelic imbalance then the ratio of the two alleles (allele1/allele2) will be equal to one. A ratio greater than one indicates an over-expression of allele1, while a ratio smaller than one indicates an over-expression of allele2.

**Figure 2:**
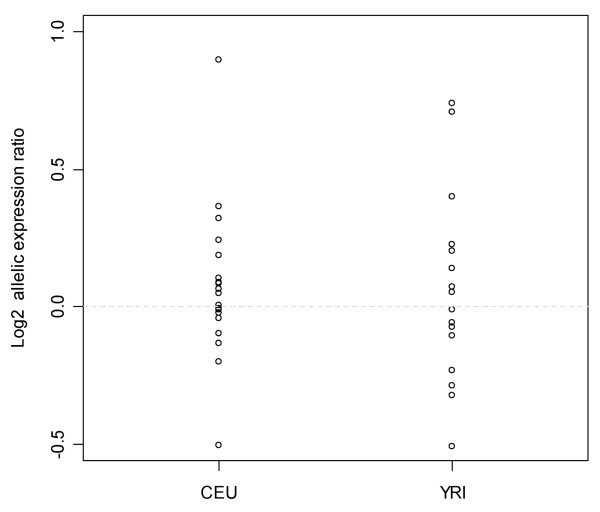
**Allele specific differences in *TLR9 *expression**. Log2 allelic expression ratio between the A allele and the G allele in individuals heterozygous for the rs352140. Represented are the results for the 21 CEU and 19 YRI individuals. The mean allelic transcript ratio for each cDNA sample was normalised to the mean allelic transcript ratio of the respective gDNA control. The horizontal dashed lines indicate the A/G allele ratio of 1.

Of the 21 CEU individuals, 17 (84%) showed no or low magnitude allelic variation (both alleles expressed nearly equally) (Figure 2). However, for three individuals, the allelic transcript A was clearly over-expressed (ratios between 1.3 and 1.87) and for one individual this allelic transcript was clearly under-expressed (average ratio of 0.5). As for the CEU population, almost all the YRI samples (80%) showed low magnitude allelic variation (Figure 2). However, three individuals showed over-expression (ratios between 1.2 and 1.67) of allele A, and one individual relatively under-expression of the same allele (average ratio of 0.7). Biological replicates from independent cultures yielded convergent results (Pearson correlation coefficient r = 0.84). Since the overall experimental variability was very low, being little influenced by variations in cell culture or other experimental techniques, these results strongly suggest that the allelic imbalances observed in the six individuals studied here represent a true biological phenomenon. As the allelic imbalance data observed was bidirectional, i.e. showing over-expression of different alleles among the cell lines, the *cis*-acting variant is likely to be in low LD with the transcript SNP [15,27].

**Figure 3:**
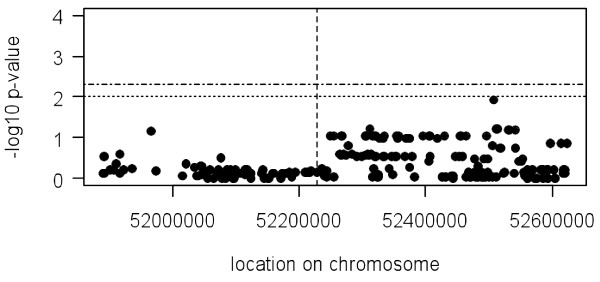
**Mapping putative *cis*-variants acting on *TLR9 *gene expression**. Allele transcript ratio mapping for 278 SNP across ~400 kb each side of the *TLR9 *gene. No association was found with any SNP surrounding the *TLR9 *gene. Y-axis represents the -log_10 _p-value and the x-axis the location in the chromosome. The horizontal dashed line indicates a nominal p-value of 0.01. The horizontal dashed and pointed line indicates the region-wide significance threshold of 1% calculated by performing 1000 permutations. The vertical dashed line indicates the position of the *TLR9 *transcribed SNP (rs352140). Each bullet represents a tested SNP. This data was obtained using the CEU data set. Similar results were obtained for the YRI.

To map potential *cis-*acting variants that might be responsible for the allelic imbalances observed in the *TLR9 *gene, the allele ratio data is correlated with haplotypes (~400 kb surrounding each side of the *TLR9 *gene) of the HapMap individuals considered using a linear regression approach (described in materials and methods) [15,24]. CEU and YRI sample sets were analysed independently. No significant associations were found between allelic imbalances and the 278 SNPs surrounding *TLR9 *gene including the promoter and intronic SNPs analysed (Figure 3). The fact that it was not possible to map any putative *cis*-variants might be because the allelic imbalances observed were caused by variants still not represented by HapMap data at the current level of SNP ascertainment or due to the reduced statistical power resulting from the small sample size used (21 CEU and 19 YRI).

Overall, while no functional polymorphism were mapped using the current HapMap genotyping data, this data shows that *TLR9 *expression is potentially modulated through *cis*-regulatory variants.

## Conclusion

To determine if genetic variation in the *TLR9 *gene influences disease outcome in malaria, a genetic association study was conducted to investigate the possible effect of *TLR9 *polymorphisms on severe malaria in two African populations. This study uses a standardized phenotype definition and study design, is the largest of its kind to date for *TLR9*, and includes both case-control and family-based data. No association was found with *TLR9 *polymorphism and severe manifestation of malaria in the Gambian cohorts and only a weak effect was observed in the Malawi case-control study. In the latter, it was not possible to adjust the potentially confounding effect of ethnicity, and possible population structure. When the more robust TDT analysis was applied to the Malawian trios, no association was observed. This analysis considered all common variants in the *TLR9 *region, but it remains possible that there are rare variants with association signals.

It is also possible that there exist *TLR9 *regulatory variants that contribute to severe malaria resistance/susceptibility that have not been mapped. It is known that regulatory polymorphisms which control gene expression can be localized hundreds of kb from the gene they influence[10,15]. Selection of candidate SNPs for association analysis close to the gene of interest is therefore likely to miss distant functional polymorphisms. One approach that will enrich potential functional polymorphism in association analysis is to use expression studies to identify SNPs that modulate gene expression. In an attempt to find putative *cis*-variants affecting *TLR9 *expression, an allele-specific expression assays was carried out in LCL from both CEU and YRI HapMap individuals. Interestingly, allelic expression imbalances were observed in both sample sets, which is reproducible in the same individuals, indicating strongly the existence of *cis*-regulation. This observation indicates clearly that different individuals might differ in the expression of *TLR9*, which can lead to different responses to the stimulus induced by malaria motifs or other pathogens. The identification of these functional genetic variants will be crucial to attest any involvement of *TLR9 *in severe malaria. The finding that *TLR9 *expression is under *cis*-regulation will strongly pave the path for further studies of infectious diseases, including the consequences of differential *TLR9 *activation.

Although evidence of *cis*-regulation of *TLR9 *expression was found, it was not possible to identify polymorphisms that correlated with the observed distortion. This is likely to be due to the incomplete polymorphism coverage of the HapMap and by the small sample sizes of the current available LCL. Increasing the number of available cell lines with abundant genotyping data or the application of new re-sequencing technologies to discover new and rare polymorphism will greatly facilitate the identification of functional variants. This work illustrates the need to upscale approaches and resources in expression analysis in the search for disease associated variants.

## Competing interests

The authors declare that they have no competing interests.

## Authors' contributions

SC coordinated the project, carried out the molecular genetic studies, statistical analysis and wrote the manuscript. JF, SA, AR, AF, JH, MD helped in the molecular genetic studies and writing the manuscript. TC performed the statistical analysis and helped writing the paper. MJ, FS, MP, MM, TT coordinated sample collection and DNA extraction. SC, KR, DK conceived the study, and participated in its design and coordination and helped to draft the manuscript.
